# CONFIDENCE treatment success: long-term real-world effectiveness and safety of ocrelizumab in Germany

**DOI:** 10.3389/fneur.2025.1564327

**Published:** 2025-05-21

**Authors:** Mathias Buttmann, Martin S. Weber, Sven G. Meuth, Sandra Blümich, Stefanie Hieke-Schulz, Petra Dirks, Julius C. Eggebrecht, Tjalf Ziemssen

**Affiliations:** ^1^Department of Neurology, Caritas Hospital, Bad Mergentheim, Germany; ^2^Department of Neurology, Institute of Neuropathology, University Medicine Göttingen, Göttingen, Germany; ^3^Fraunhofer-Institute for Translational Medicine and Pharmacology ITMP, Göttingen, Germany; ^4^Department of Neurology, University Clinic Düsseldorf, Heinrich-Heine-University Düsseldorf, Düsseldorf, Germany; ^5^Roche Pharma AG, Grenzach-Wyhlen, Germany; ^6^F. Hoffmann-La Roche AG, Basel, Switzerland; ^7^Center of Clinical Neuroscience, Department of Neurology, Faculty of Medicine and University Hospital Carl Gustav Carus, TUD Dresden University of Technology, Dresden, Germany

**Keywords:** Relapsing multiple sclerosis, humanized monoclonal anti-CD20 antibody, neurodegenerative diseases, non-interventional study (NIS), ocrelizumab, real-world cohort, treatment success, effectiveness

## Abstract

**Background:**

Early high-efficacy treatment for people with relapsing multiple sclerosis (pwRMS) may provide better long-term outcomes compared with the escalation strategy. In this study, we present an analysis of treatment success and safety from the CONFIDENCE study in a real-world cohort of pwRMS treated with ocrelizumab in different treatment lines for up to 5.5 years.

**Methods:**

The ongoing German non-interventional post-authorization safety study, CONFIDENCE (ML39632, EUPAS22951), evaluates the long-term safety and effectiveness of therapy in pwMS newly treated with ocrelizumab or other disease-modifying therapies for up to 10 years. This analysis presents CONFIDENCE treatment success (proportion of people with no clinical disease activity measured by relapse or disease progression and no treatment discontinuation due to adverse event [AE] or lack of therapeutic effectiveness), confirmed disability progression (CDP), annualized relapse rates, and safety in pwRMS stratified by the number of previous MS therapies (PMSTs).

**Results:**

At the data cutoff (11 October 2023), the full analysis set included 2,261 pwRMS treated with ≥1 dose of ocrelizumab. At baseline, the mean age (SD) of the participants was 41.16 (11.39) years (treatment-naïve, 39.19 [12.95] years; ≥3 PMSTs, 42.80 [10.08] years), and the mean Expanded Disability Status Scale (EDSS) score was 3.08 (1.86) (treatment-naïve, 2.37 [1.54]; ≥3 PMSTs, 3.57 [1.90]). Overall, 58.4% of pwRMS with continuous treatment achieved CONFIDENCE treatment success from baseline until year 5 (74.0 and 50.3% of pwRMS with 0 and ≥3 PMSTs). The main reasons for not achieving CONFIDENCE treatment success were relapse and CDP, while treatment discontinuation due to AEs or lack of effectiveness played a minor role. The proportion of pwRMS with AEs did not increase with longer treatment duration and tended to be higher with more PMSTs. The spectrum of AEs was similar across treatment lines, and no new or unexpected AEs were observed.

**Conclusion:**

CONFIDENCE treatment success remained high over 5 years of ocrelizumab treatment, even among people with RMS (pwRMS) with a higher number of PMSTs. Only a small proportion of pwRMS discontinued treatment due to AEs. These results support early intervention with high-efficacy ocrelizumab treatment to optimize long-term outcomes for pwRMS.

**Trial registration:**

https://catalogues.ema.europa.eu/node/3142/administrative-details, identifiers ML39632 and EUPAS22951.

## 1 Introduction

Multiple sclerosis (MS) is an immune-mediated neurodegenerative disease of the central nervous system (CNS) that clinically starts with relapses (relapsing MS, RMS) or with a steady decline from onset (primary progressive MS, PPMS) (1). Ocrelizumab (Ocrevus^®^) is the first drug approved for the disease-modifying treatment (DMT) of both RMS and PPMS. In the OPERA I and II trials, people with RMS (pwRMS) treated with ocrelizumab experienced significantly reduced disease activity and progression, along with a similar safety profile compared to interferon β-1a (2). This effect was maintained during follow-up for up to 10 years (3, 4). Effectiveness and safety for most common adverse events (AEs), including infections and infestations in ocrelizumab-treated pwMS, have also been described in the real-world setting, confirming the favorable benefit–risk profile demonstrated in pivotal trials (5–22).

Accumulating evidence favors early intervention with high-efficacy treatments, including anti-CD20 therapeutics (23–25). With the introduction of highly effective MS treatments, the aim of achieving no evidence of disease activity (NEDA) has been established as a desirable outcome (26). NEDA is defined as the absence of relapses, disability progression, and focal-inflammatory magnetic resonance imaging (MRI) measures of disease activity (NEDA-3) or, additionally, of no brain volume loss above the levels of healthy controls (NEDA-4) (26). However, due to the limited availability of the required technological infrastructure and trained staff, NEDA assessment is currently not broadly applicable in routine clinical practice (26).

The ongoing German non-interventional post-authorization safety study (NI-PASS) CONFIDENCE (ML39632, EUPAS22951) evaluates the long-term safety and effectiveness of ocrelizumab and other DMTs (i.e., alemtuzumab, cladribine, dimethyl fumarate, fingolimod, natalizumab, or teriflunomide) in a real-world MS population for up to 10 years (27).

This analysis assesses CONFIDENCE treatment success and safety of ocrelizumab in pwRMS enrolled in CONFIDENCE, who completed treatment for up to a maximum of 5.5 years and stratified by the number of prior MS-specific therapies (PMSTs).

## 2 Materials and methods

### 2.1 Study design

The German NI-PASS CONFIDENCE analyzes long-term safety and effectiveness outcomes in pwMS newly treated with ocrelizumab or other selected DMTs (alemtuzumab, cladribine, dimethyl fumarate, fingolimod, natalizumab, and teriflunomide). The decision for treatment prescription was made prior to and independently of their participation in this study. Follow-up occurs every ~6 months for up to 10 years regardless of treatment discontinuation. Full details on the CONFIDENCE study design and inclusion/exclusion criteria were published previously (8, 27). Recruitment started in April 2018 and was completed in March 2022.

### 2.2 Trial registration and ethics statement

CONFIDENCE was registered on 6 March 2018 under the EUPAS Register number EUPAS22951.^1^ The independent ethics committee at the Technical University Dresden has provided initial professional advice (Ethikkommission an der Technischen Universität Dresden, Germany; 12 February 2018 and 10 April 2019; reference EK 62022018). Obtaining further ethical approval was the individual responsibility of the participating physicians.

### 2.3 Endpoints

#### 2.3.1 Effectiveness

In CONFIDENCE, treatment success was assessed cumulatively as a composite endpoint for a total of 1–5 years of treatment in a landmark analysis (Supplementary Figure 1). Only people still on ocrelizumab treatment after completion of 1, 2, 3, 4, and 5 years of treatment were considered for the respective analyses. CONFIDENCE treatment success was defined as the proportion of people with no clinical disease activity measured by relapse or disease progression, as well as no treatment discontinuation due to AEs or lack of therapeutic effectiveness. Clinical disease activity was measured using clinical data from the database, with 24-week confirmed disability progression (24-week CDP) as the primary measure. Treatment discontinuation due to AEs or lack of therapeutic effectiveness was assessed by the physician’s judgment and could be selected upon treatment switch, independent of clinical data. CONFIDENCE treatment success was considered to not have been reached if any of the criteria were not met. Multiple reasons for not reaching CONFIDENCE treatment success might apply per pwRMS.

Twenty-four-week CDP was defined as an increase in the Expanded Disability Status Scale (EDSS) score of: ≥1.0 points from the baseline EDSS score when the baseline score was ≤5.5; ≥0.5 points from the baseline EDSS score when the baseline score was >5.5. CDP was confirmed when the increase in EDSS was confirmed at a regularly scheduled visit at least 24 weeks after the initial documentation of neurological worsening and no relapses occurred in the meantime. The proportion of pwRMS with 24-week CDP only, with relapse only, and with both (CDP plus relapse) during the respective observation time (up to year 1, 2, 3, 4, and 5) was determined. In addition, 24-week CDP was analyzed using the Kaplan–Meier method.

Annualized relapse rate (ARR) was calculated separately for each of the treatment years, which was standardized to 12 months. ARR was defined as the total number of protocol-defined relapses per pwRMS since the start of treatment.

#### 2.3.2 Safety

Safety data were recorded by system organ class (SOC) and preferred term (PT) of the Medical Dictionary for Regulatory Activities (MedDRA; version 26.0) over a maximum of 5.5 years of study observation, representing a total of 7,743.41 person-years (PY) on ocrelizumab treatment. The safety analyses considered all pwRMS who completed ocrelizumab treatment for the respective evaluated year. Endpoints include the proportions of pwRMS with AEs, serious AEs (SAEs), and infections and infestations, including serious events.

### 2.4 Statistical analysis

This analysis comprises pwRMS (safety analysis set; SAS) who received ≥1 dose of ocrelizumab (data cutoff 11 October 2023). The full analysis set (FAS) used for effectiveness analyses comprised pwRMS of the SAS with ≥1 follow-up visit after therapy start. Descriptive statistics were used for all continuous variables, with a 95% confidence interval (CI) where applicable. Data are presented for the entire cohort of pwRMS and stratified by the number of PMSTs: 0 (treatment-naïve), 1, 2, and ≥3 PMSTs. Baseline characteristics are presented as mean ± standard deviation (SD).

CONFIDENCE treatment success is described as the proportion of pwRMS (FAS) achieving treatment success after 1, 2, 3, 4, and 5 years of follow-up. Only pwRMS still on treatment at the end of a specific analysis year were considered. Multivariate logistic regression analyses were performed to evaluate associations between subgroup variables and CONFIDENCE treatment success, including EDSS at baseline, number of PMSTs, age, last MS-specific pre-treatment, and sex as independent variables, and CONFIDENCE treatment success as a dependent variable. Odds ratios (ORs) are presented with 95% CI according to the Wald method.

ARRs were calculated as the mean number of relapses per group since the start of treatment, standardized to 365.25 days, and different treatment durations for each pwRMS were considered as individual log-transformed exposure time for the computation of relapse rates.

CDP was analyzed as survival probability using the Kaplan–Meier method and is presented as time to event.

Time-adjusted analyses of the incidence of AEs, SAEs, and infections and infestations were performed based on the total number of events per SOC-PT divided by the number of PY (calculated as the person’s exposure to treatment and/or the person’s duration of observation).

Statistical analysis was performed using SAS software, version 9.4 (TS1M5) of SAS System for Windows (SAS Institute Inc., Cary, NC, USA).

## 3 Results

### 3.1 Baseline characteristics

As of data cutoff, the FAS included 2,261 pwRMS and the SAS included 2,267 pwRMS (7,743.41 PY). Outcome analyses on pwRMS in the FAS are limited to those who completed ≥1 year of treatment (*n* = 2,137). Baseline characteristics of the FAS are shown in Table 1. The mean (SD) observation time since enrolment was 3.21 (1.31) years. Overall, the mean age at baseline was 41.16 (11.39) years, which increased numerically with an increasing number of PMSTs from 39.19 (12.95) years (treatment-naïve, 0 PMSTs) to 42.80 (10.08) years (≥3 PMSTs). The overall mean EDSS at baseline was 3.08 (1.86), which increased numerically with a higher number of PMSTs (2.37 [1.54] vs. 3.57 [1.90] for 0 vs. ≥3 PMSTs). When stratified by PMSTs (0, 1, 2, and ≥3 PMSTs), the mean time since the first MS symptoms was higher with an increasing number of PMSTs (3.87 [6.10] vs. 15.23 [7.84] years for 0 vs. ≥3 PMSTs). Overall, 72.4% of pwRMS were women.

**Table 1:** Baseline characteristics (full analysis set).

Characteristic	Overall (*n* = 2,261; 100%)	Treatment-naïve (0 PMSTs) (*n* = 403; 17.8%)	1 PMST (*n* = 546; 24.1%)	2 PMSTs (*n* = 534; 23.6%)	≥3 PMSTs (*n* = 778; 34.4%)
Mean age, years (SD)	41.16 (11.39)	39.19 (12.95)	39.64 (11.61)	41.82 (11.32)	42.80 (10.08)
Female, n (%)	1,637 (72.4)	274 (68.0)	377 (69.0)	383 (71.7)	603 (77.5)
Mean time to baseline since, years (SD)					
First MS symptoms	10.76 (8.76)	3.87 (6.10)	8.73 (8.83)	11.44 (7.64)	15.23 (7.84)
Diagnosis	8.97 (7.83)	2.18 (5.00)	6.61 (7.01)	9.81 (6.72)	13.57 (7.09)
Mean EDSS (SD)	3.08 (1.86)	2.37 (1.54)	2.73 (1.76)	3.23 (1.87)	3.57 (1.90)
Last MS-specific therapy before ocrelizumab, n (%)					
Dimethyl fumarate	304 (13.4)	-	120 (22.0)	86 (16.1)	98 (12.6)
Fingolimod	422 (18.7)	-	49 (9.0)	166 (31.1)	207 (26.6)
Interferon or glatiramer acetate	360 (15.9)	-	213 (39.0)	63 (11.8)	84 (10.8)
Natalizumab	314 (13.9)	-	50 (9.2)	103 (19.3)	161 (20.7)
Other	861 (38.1)	-	114 (20.9)	116 (21.7)	228 (29.3)
Mean observation time, years (SD)	3.21 (1.31)	3.07 (1.27)	3.31 (1.28)	3.21 (1.32)	3.23 (1.35)

EDSS, Expanded Disability Status Scale; MS, multiple sclerosis; PMST, prior MS-specific therapy; SD, standard deviation.

### 3.2 CONFIDENCE treatment success

**Figure 1:**
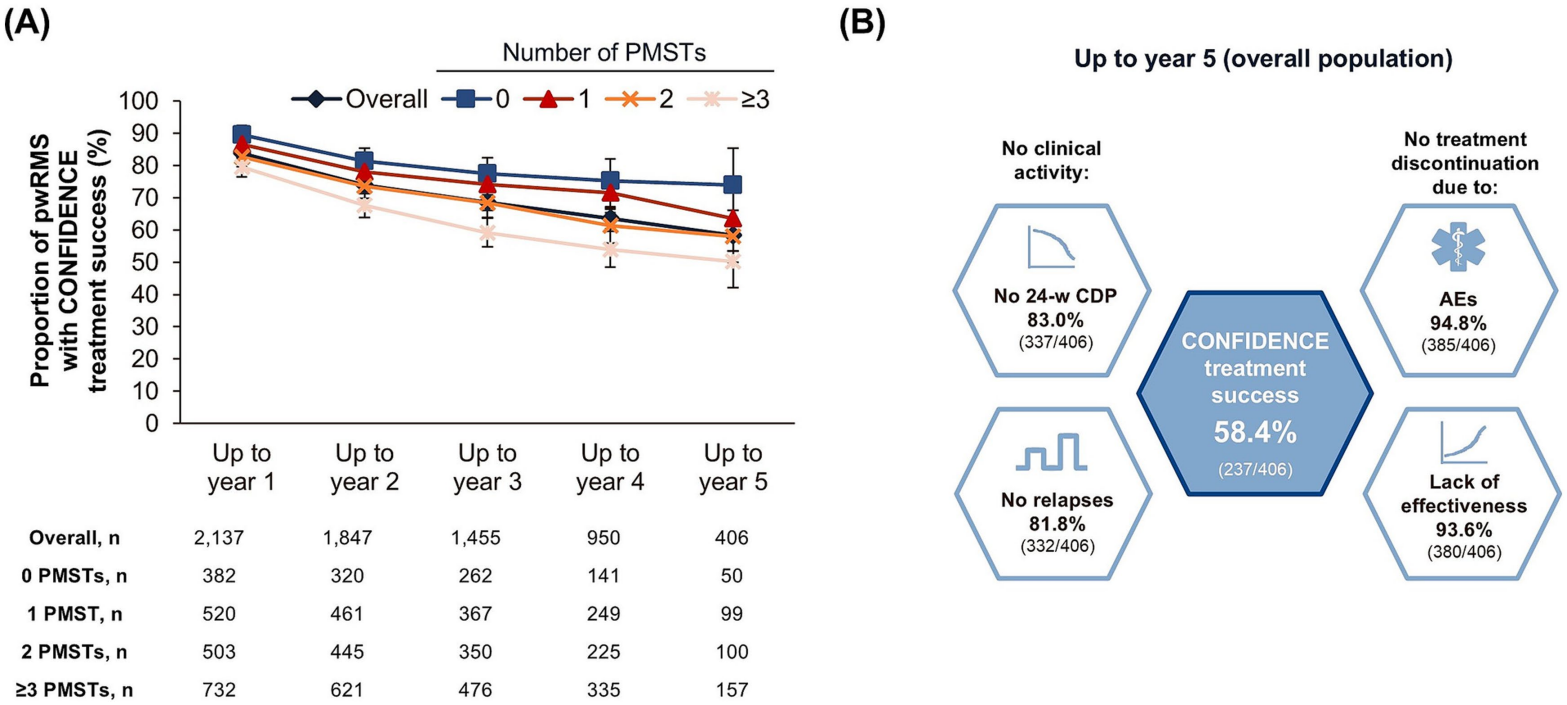
CONFIDENCE treatment success **(A)** by number of prior MS-specific therapies over a maximum of 5 years of ocrelizumab therapy and **(B)** for the overall cohort from baseline to year 5 who completed year 5 (landmark analysis; FAS). **(A)** Error bars represent 95% confidence intervals. **(B)** The outer hexagons describe the individual components of CONFIDENCE treatment success, which is illustrated in the inner hexagon. AE, adverse event; 24-w CDP, 24-week confirmed disability progression; FAS, full analysis set; MS, multiple sclerosis; PMST, prior MS-specific therapy; pwRMS, people with relapsing MS.

**Figure 2:**
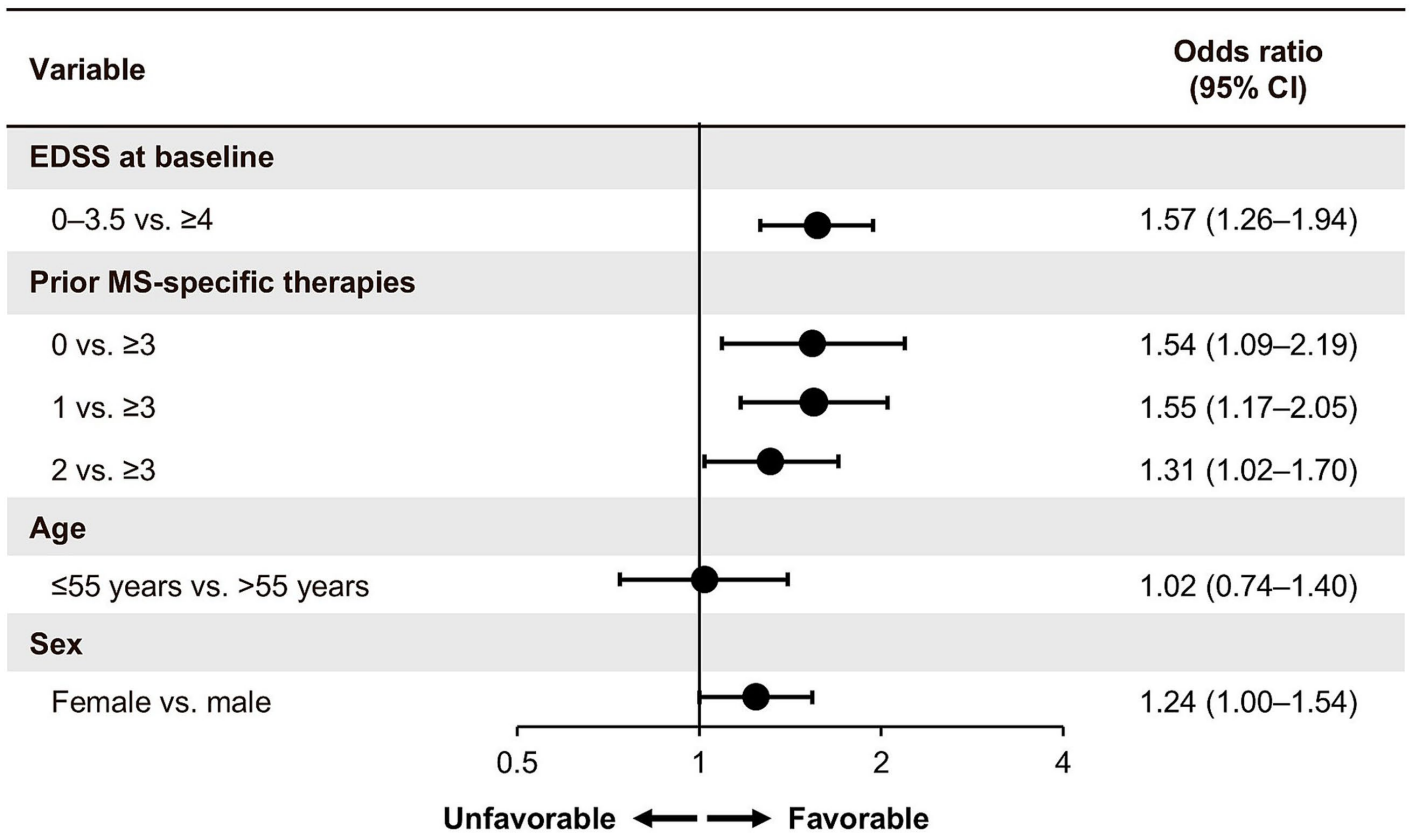
Multivariate logistic regression analysis of CONFIDENCE treatment success (FAS). pwRMS with available data were included (*n* = 1,907). EDSS, Expanded Disability Status Scale; CI, confidence interval; FAS, full analysis set; MS, multiple sclerosis; PMST, prior MS-specific therapy.

From baseline to the end of treatment year 1, 2, 3, 4, and 5, 83.8% (1,791/2,137), 74.0% (1,367/1,847), 68.5% (996/1,455), 63.5% (603/950), and 58.4% (237/406) of pwRMS, respectively, achieved CONFIDENCE treatment success in the overall population (Figures 1A,B). From baseline to the end of year 5, 74.0% (37/50), 63.6% (63/99), 58.0% (58/100), and 50.3% (79/157) of pwRMS with 0, 1, 2, and ≥3 PMSTs achieved CONFIDENCE treatment success (Figure 1A). Multivariate logistic regression analysis (*n* = 1,907) showed that a baseline EDSS of 0–3.5 was significantly associated with a higher likelihood of achieving CONFIDENCE treatment success compared to a baseline EDSS of ≥4 (OR 1.57, 95% CI 1.26–1.94) (Figure 2). Treatment-naïve pwRMS were more likely to achieve CONFIDENCE treatment success than pwRMS treated with ≥3 PMSTs (0 vs. ≥3 PMSTs, OR 1.54, 95% CI 1.09–2.19) (Figure 2). Women were more likely to achieve CONFIDENCE treatment success than men (OR 1.24, 95% CI 1.00–1.54). No significant association was observed between age at baseline and CONFIDENCE treatment success (Figure 2).

**Figure 3:**
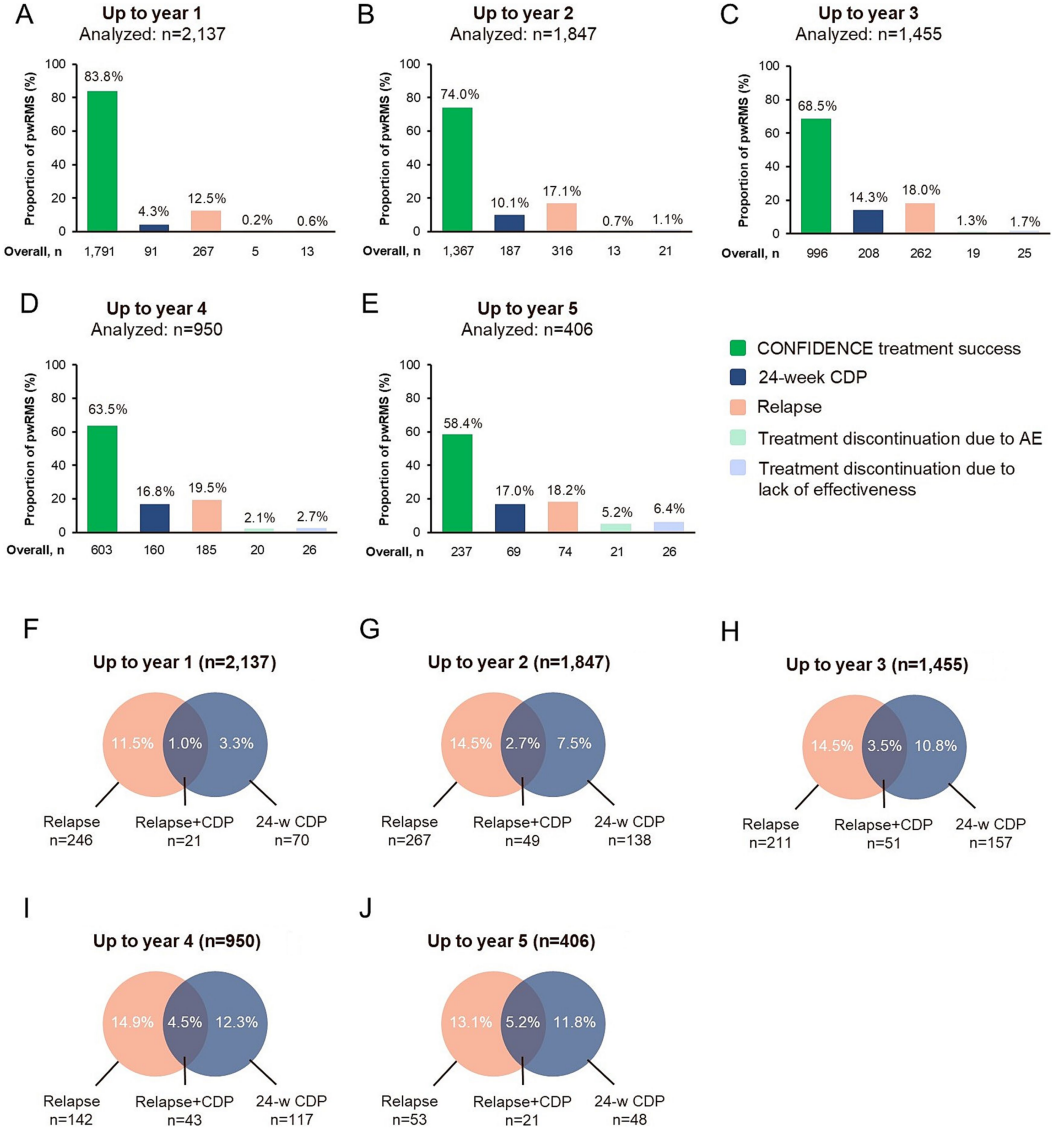
CONFIDENCE treatment success per year (landmark analysis; FAS). **(A–E)** CONFIDENCE treatment success and reasons for not reaching CONFIDENCE treatment success in the overall population up to **(A)** year 1, **(B)** year 2, **(C)** year 3, **(D)** year 4, and **(E)** year 5. Multiple answers were possible per pwRMS. **(F–J)** Percentages of pwRMS with no CONFIDENCE treatment success due to relapse only, 24-week CDP only, or both relapse and 24-week CDP in the overall population relative to the number of pwRMS analyzed for CONFIDENCE treatment success from **(F)** year 1, **(G)** year 2, **(H)** year 3, **(I)** year 4, and **(J)** year 5. AE, adverse event; 24-w CDP, 24-week confirmed disability progression; FAS, full analysis set; MS, multiple sclerosis; pwRMS, people with relapsing MS.

**Figure 4:**
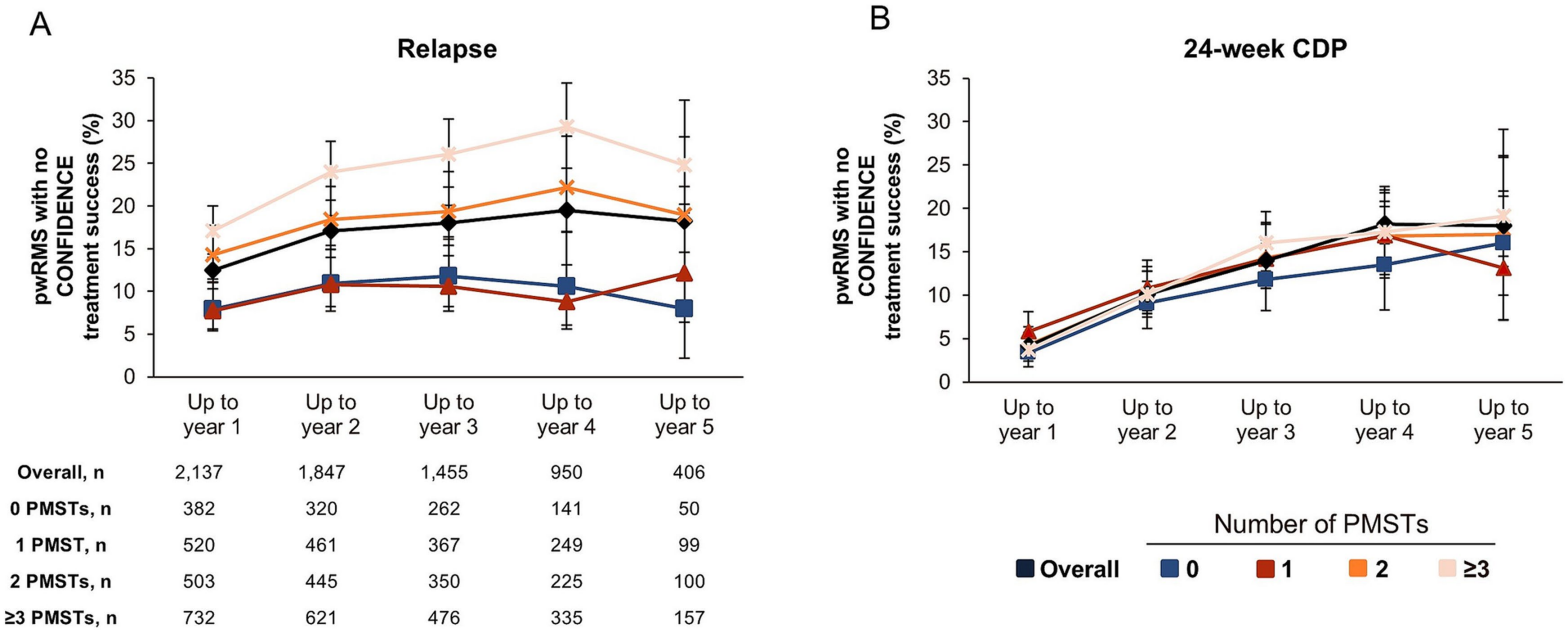
Reasons for not reaching CONFIDENCE treatment success stratified by the number of PMSTs (landmark analysis; FAS). **(A)** Percentage of pwRMS with no CONFIDENCE treatment success due to relapse up to the respective treatment year over a maximum of 5 years of ocrelizumab therapy. **(B)** Percentage of pwRMS with no CONFIDENCE treatment success due to 24-w CDP up to the respective treatment year over a maximum of 5 years of ocrelizumab therapy. Percentages of pwRMS with no CONFIDENCE treatment success are relative to the number of pwRMS analyzed for CONFIDENCE treatment success up to the respective year. Error bars represent 95% confidence intervals. 24-w CDP, 24-week confirmed disability progression; FAS, full analysis set; MS, multiple sclerosis; PMST, prior MS-specific therapy; pwRMS, people with relapsing MS.

The main reasons for not achieving CONFIDENCE treatment success for the overall population were relapse [12.5% (267/2,137), 17.1% (316/1,847), 18.0% (262/1,455), 19.5% (185/950), and 18.2% (74/406) for up to 1, 2, 3, 4, and 5 years of treatment, respectively] and 24-week CDP [4.3% (91/2,137), 10.1% (187/1,847), 14.3% (208/1,455), 16.8% (160/950), and 17.0% (69/406) for up to 1, 2, 3, 4, and 5 years of treatment, respectively] (Figures 3A–E). A comparably small proportion of pwRMS did not reach CONFIDENCE treatment success because of treatment discontinuation due to AEs or due to the lack of effectiveness (0.2, 0.7, 1.3, 2.1, 5.2%, and 0.6, 1.1, 1.7, 2.7, 6.4%, respectively, up to treatment years 1, 2, 3, 4, and 5) (Figure 3A–E; for a summary of reasons for premature study termination please see Supplementary Table 1). The proportion of pwRMS with both relapse and 24-week CDP was lower than the proportion of pwRMS with CDP or relapse only (1.0, 2.7, 3.5, 4.5, and 5.2% for up to 1, 2, 3, 4, and 5 years of treatment, respectively) (Figures 3F–J). Relapse as a reason for not reaching CONFIDENCE treatment success tended to increase with the number of PMSTs. The proportion of pwRMS experiencing CDP as a reason for not achieving CONFIDENCE treatment success was similar in all subgroups and increased with treatment years (Figure 4A). However, treatment-naïve pwRMS tended to have less 24-week CDP (Figure 4B).

### 3.3 ARR and CDP

**Figure 5:**
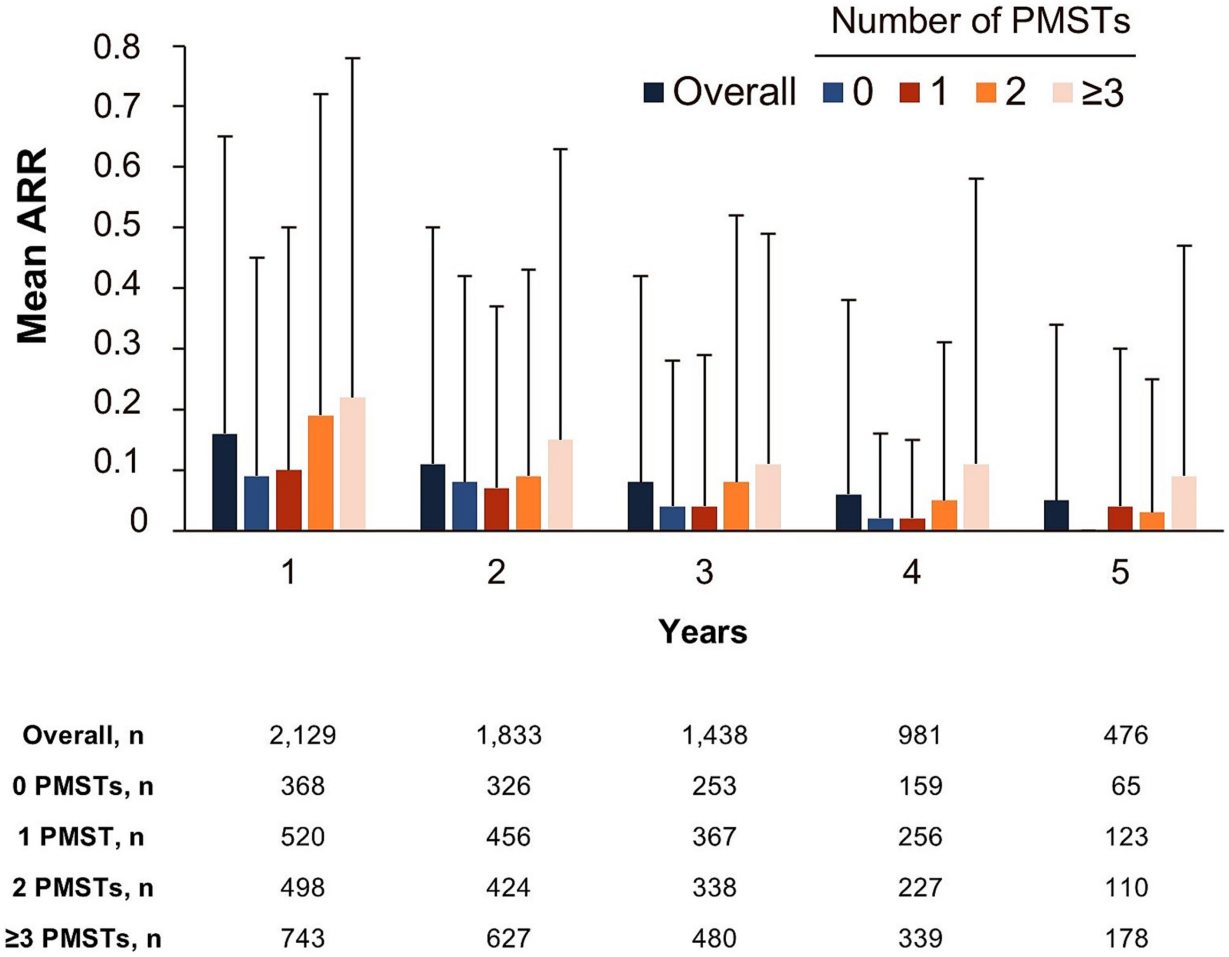
Annualized relapse rate by the number of PMSTs over a maximum of 5 years of ocrelizumab therapy (FAS). Error bars represent the standard deviation. ARR, annualized relapse rate; FAS, full analysis set; MS, multiple sclerosis; PMST, prior MS-specific therapy.

**Figure 6:**
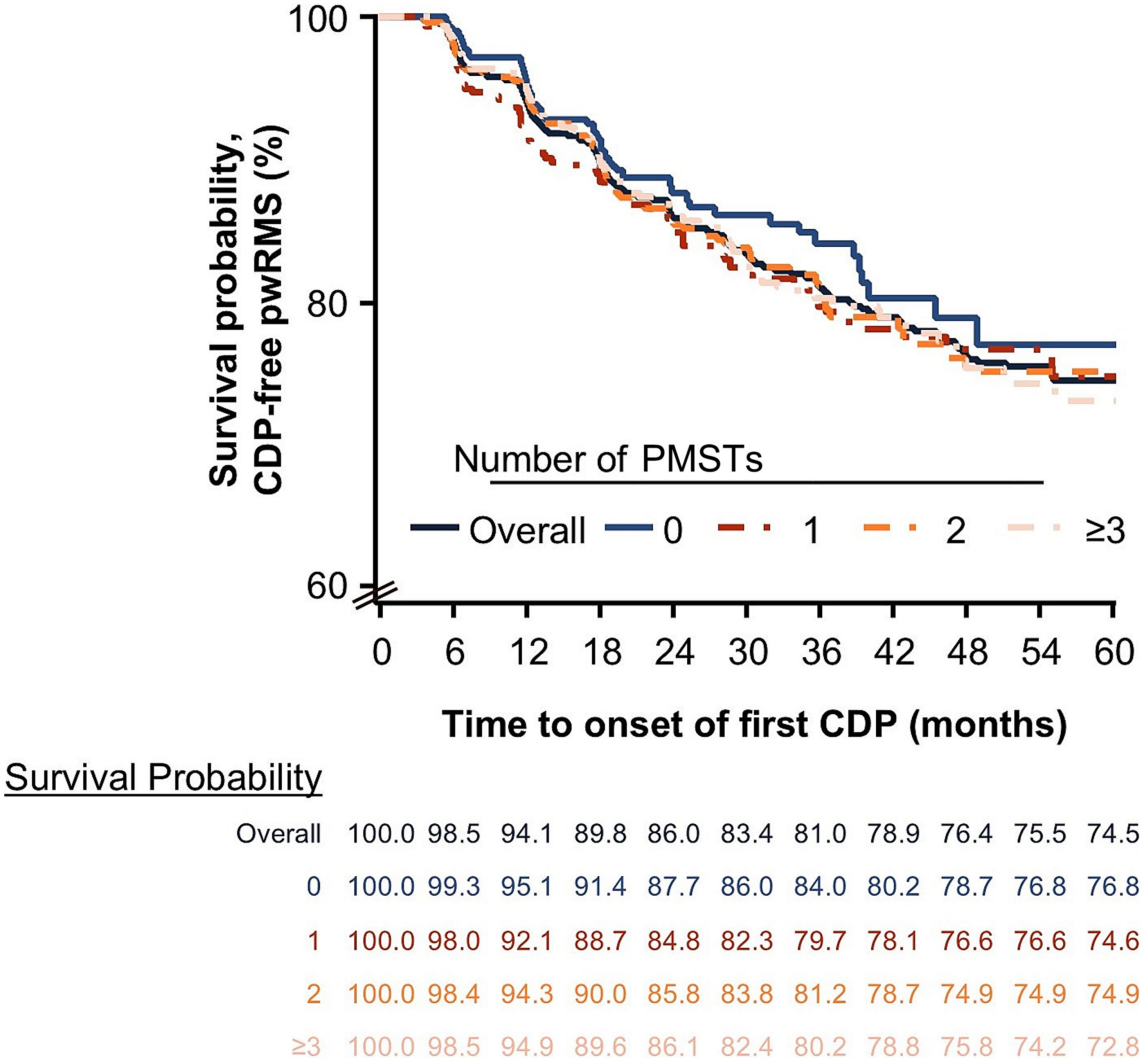
Time to onset of 24-week CDP by the number of PMSTs over a maximum of 5 years of ocrelizumab therapy (FAS). Survival probability was determined using the Kaplan–Meier method. The y-axis only covers 60–100% for better readability. CDP, confirmed disability progression; FAS, full analysis set; MS, multiple sclerosis; PMST, prior MS-specific therapy; pwRMS, people with relapsing MS.

The overall mean (SD) ARR was 0.11 (0.31) (*n* = 2,140). The mean ARR per year decreased with treatment duration (0.16 [0.49], 0.11 [0.39], 0.08 [0.34], 0.06 [0.32], and 0.05 [0.29] for years 1, 2, 3, 4, and 5, respectively). The decrease in ARR over treatment duration was independent of the treatment line (Figure 5). Generally, pwRMS treated with ocrelizumab in an early line tended to have lower ARRs compared with pwRMS with a higher number of PMSTs (Figure 5). The analysis of time to onset of first CDP showed that the probability of remaining CDP-free over 5 years was 74.5% in the overall population (*n* = 1,907), with a slightly higher probability in first-line pwRMS (Figure 6).

### 3.4 Safety

**Figure 7:**
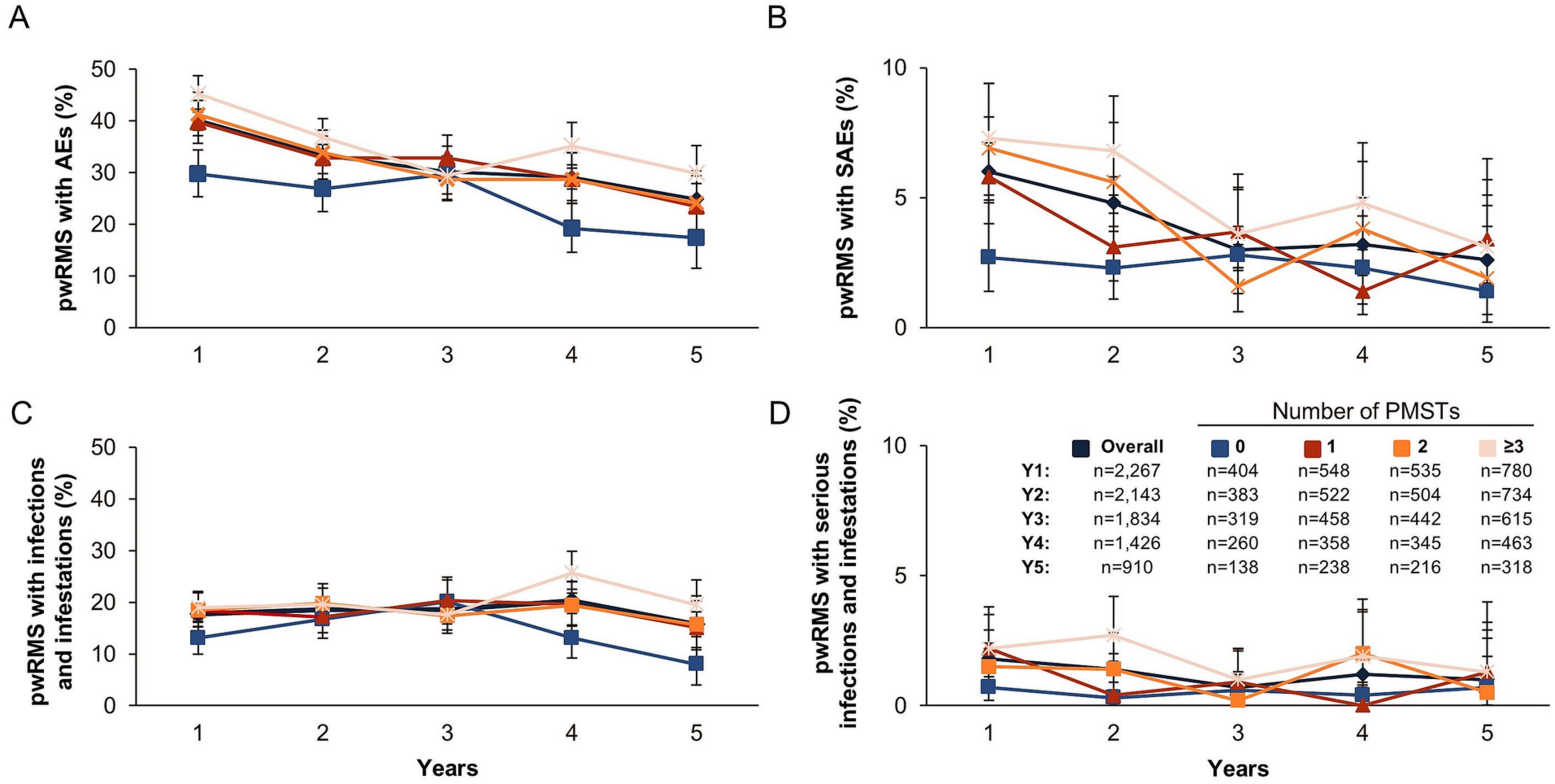
Safety outcomes by the number of PMSTs over a maximum of 5 years of ocrelizumab therapy (SAS). Percentages of pwRMS with **(A)** adverse events, **(B)** serious adverse events, **(C)** infections and infestations, and **(D)** serious infections and infestations by treatment year. Error bars represent 95% confidence intervals. AE, adverse event; MS, multiple sclerosis; SAS, safety analysis set; SAE, serious adverse event; pwRMS, people with relapsing MS; Y, year.

Safety is described for the overall RMS population (SAS; *n* = 2,267; 7,743.41 PY), including pwRMS with up to 5.5 years of ocrelizumab treatment. Among the analyzed SAS population, 1,476 pwRMS treated with ocrelizumab experienced 77.5 events/100 PY, and 331 pwRMS experienced 7.3 serious events/100 PY. PwRMS with 0, 1, 2, and ≥3 PMSTs experienced 56.7, 76.2, 77.9, and 88.7 AEs/100 PY, respectively, and 3.9, 6.7, 7.0, and 9.5 SAEs/100 PY. The proportion of pwRMS with AEs and SAEs remained stable with an increasing number of treatment years and tended to be lower with fewer PMSTs (Figures 7A, B). No new or unexpected AEs were observed. Overall, the AE rate did not increase with extended treatment duration, and the types of AEs were similar between different treatment lines (data not shown).

The proportion of pwRMS with infections and infestations and serious infections and infestations tended to be lower with fewer PMSTs (Figures 7C,D). Of all pwRMS (SAS population), 997 pwRMS experienced 28.8 events/100 PY infections and infestations (2,229 events) over a treatment period of 5 years. The three most common infections and infestations were COVID-19 (8.2 events/100 PY), nasopharyngitis (4.8 events/100 PY), and urinary tract infection (4.0 events/100 PY).

Overall, the proportion of pwRMS that experienced infections and infestations (17.7, 18.6, 18.6, 20.3, and 15.7% at years 1, 2, 3, 4, and 5, respectively) and serious infections and infestations (1.8, 1.4, 0.7, 1.2, and 1.0% at years 1, 2, 3, 4, and 5, respectively) did not increase with extended treatment duration (Figures 7C,D). The three most common serious infections and infestations were COVID-19 (0.5%), pneumonia (0.5%), and diverticulitis (0.2%) in treatment-naïve pwRMS; COVID-19 (0.9%), pneumonia (0.5%), and urinary tract infection (0.5%) in pwRMS with 1 PMST; COVID-19 (1.5%), pneumonia (0.6%), and pyelonephritis (0.6%) in pwRMS with 2 PMSTs; and COVID-19 (1.3%), urinary tract infection (1.3%), and pneumonia (0.9%) in pwRMS with ≥3 PMSTs (the cases of pneumonia were not COVID-19-associated). Overall, 12 fatal treatment-emergent AEs (TEAEs) occurred in 7 pwRMS (0.3%; 0.2 events/100 PY) (Supplementary Table 2); 7 fatal TEAEs were considered unrelated to ocrelizumab by the respective treating physician, and 5 fatal TEAEs were not assessed for potential relation to ocrelizumab. The main reported reasons for death were “cardiac disorders” and “general disorders and administration site conditions” (Supplementary Table 2). One COVID-19-related fatality was reported in 1 pwRMS, which was assessed to be unrelated to ocrelizumab by the treating physician. Nevertheless, a causal relationship between anti-CD20 therapy and a fatal course of COVID-19 cannot be fully excluded.

## 4 Discussion

In the era of high-efficacy treatment, finding the optimal treatment strategy considering effectiveness and safety parameters presents a challenge. Long-term observational studies in real-world settings provide valuable complementary information to randomized controlled trials, support routine clinical practice, and help optimize treatment decisions (28). In the present study, we present real-world data from the CONFIDENCE study on treatment success and safety in pwRMS who received ocrelizumab over a maximum of 5.5 years.

### 4.1 Baseline characteristics

PwRMS enrolled in CONFIDENCE were on average older, had a higher EDSS at baseline, and a higher number of PMSTs than pwRMS in the pivotal trials (2). This finding is in line with baseline characteristics from several previously reported real-world cohorts treated with ocrelizumab or other high-efficacy treatments (11, 29–31), thus reflecting a representative real-world cohort.

### 4.2 CONFIDENCE treatment success

With the increasingly complex therapeutic landscape in MS, the assessment of treatment success, taking into account both effectiveness and safety parameters, is becoming increasingly important for well informed treatment decisions (32). Although NEDA has become a recognized therapeutic goal and clinical endpoint in controlled trials, its use under real-world conditions remains challenging (33). Accordingly, we defined CONFIDENCE treatment success as no clinical activity (relapse or CDP) and no treatment discontinuation due to AEs or lack of therapeutic effectiveness. This status was achieved by the majority (58.4%; 237/406) of pwRMS over a treatment duration of 5 years and was most pronounced in pwRMS with no PMSTs (74.0%; 37/50). Treatment-naïve pwRMS were more likely to achieve CONFIDENCE treatment success and demonstrate a greater response to ocrelizumab than pwRMS treated with 1, 2, or ≥3 PMSTs. This could be due to the different clinical course of each subgroup related to the natural progression of the disease over time, illustrated in this study by the difference in mean time since diagnosis for each subgroup. The observation of an increased likelihood of achieving CONFIDENCE treatment success with a lower number of PMSTs was also evident in the open-label extension of ocrelizumab phase III trials, demonstrating a sustained beneficial effect in pwRMS previously treated with ocrelizumab (3, 4). Another factor that may impact CONFIDENCE treatment success is the EDSS at baseline. In the present study, it has been observed that an EDSS of <3.5 at baseline was associated with a higher likelihood of achieving CONFIDENCE treatment success, and comparable effects were shown in previous trials (5). This finding is in line with the concept of a two-stage disability progression in MS, characterized by a predominantly inflammatory environment in the first phase of MS (EDSS 0–3), followed by increased neurodegenerative processes independent of inflammation. This results in an optimal window of opportunity for treatment with anti-inflammatory drugs in the early disease course, where an EDSS of 3 has not been exceeded (34).

The effect of sex on CONFIDENCE treatment success was assessed, as it is known to impact the incidence and severity of autoimmune neurodegenerative diseases such as MS (35). We found that women were slightly more likely to achieve CONFIDENCE treatment success than men, which may be based on sex differences in innate and adaptive immunity (36).

Approximately 40% of pwRMS did not reach CONFIDENCE treatment success up to year 5 due to mostly clinical parameters (relapses and CDP). The concomitant increase in CDP and decrease in ARR over time can reflect the well described effect of ocrelizumab on the control of acute relapse activity and again indicates the neurodegenerative processes leading to the so-called progression independent of relapse activity (PIRA) (37). PIRA, as a key component driving disease accumulation in RMS, was also described in the ocrelizumab pivotal trials, in which a reduction was shown by ocrelizumab compared to interferon *β*-1a (37). However, since our analysis does not provide the exact time point of when an event occurred, a potential (temporal) association between relapse and progression cannot be evaluated, and a potential bias resulting from pwRMS with suboptimal relapse control who might have discontinued treatment also needs to be considered. It should also be noted that a gradual increase in CDP and a decrease in ARR may reflect the natural course of the disease.

Treatment discontinuation due to AEs or lack of effectiveness contributed to the loss of CONFIDENCE treatment success only to a small extent, reinforcing the well-described long-term effectiveness of ocrelizumab as well as its manageable and well tolerated safety profile (3, 4).

### 4.3 Safety

The safety profile of DMTs is a decisive parameter when making treatment decisions. Highly effective DMTs are often perceived as therapies with an increased safety risk, which is the key barrier to their early use. Overall, the presented ocrelizumab safety data are consistent with a previous CONFIDENCE interim analysis and ocrelizumab long-term integrated pooled analysis (8, 9) as well as with reported data in pivotal clinical trials (2). No new or unexpected AEs were observed over a treatment period of up to 5.5 years. The proportion of pwRMS with AEs tended to be higher with a higher number of PMSTs. Apart from a potential effect of previous immunotherapies, the potential for AEs might be driven by the older age and longer disease duration of these pwRMS (38). The proportion of pwRMS with AEs did not increase with increased treatment duration of up to 5.5 years, and the spectrum of AEs was similar across treatment lines. No increase was observed in the proportion of pwRMS who experienced infections and infestations (including serious events), although a peak was noted at year 4. This increase might be attributed to the high number of COVID-19 infections at that time point. Interestingly, in a previous study investigating COVID-19 outcomes in ocrelizumab-treated pwMS from CONFIDENCE, it was observed that, out of the 826 participants investigated, the majority (>90%) had received a COVID-19 vaccination (39). In light of this finding, the high number of COVID-19 infections may be demonstrative of the impact of anti-CD20 therapy on the humoral immune response under vaccination, while the cellular immune response is maintained (40, 41). Case severity was not analyzed in these cases.

## 5 Strengths and limitations

As a long-term study in a real-world setting, CONFIDENCE is prone to the drawbacks inherent to non-interventional studies (e.g., potential enrollment and channeling biases between cohorts). However, only pwMS newly treated with ocrelizumab (or other DMTs) were enrolled in CONFIDENCE to minimize limitations and biases associated with long-term real-world studies (such as healthy user bias and depletion of susceptibles). CONFIDENCE treatment success was assessed in a landmark analysis, which is prone to bias due to a decrease in sample size with an increase in observation time. The limited availability of MRI data excludes the important part of subclinical disease activity in the investigation of CONFIDENCE treatment success. However, the present assessment considers additional components such as factors leading to treatment discontinuation that are relevant for treatment decisions in clinical practice (26). Due to the lack of a comparison group, no direct conclusions could be drawn about the treatment effect of ocrelizumab. While women were slightly more likely to achieve CONFIDENCE treatment success compared to men, no specific sex-related outcomes or conclusions can be drawn as the present analysis was not designed to detect differences between these treatment groups. As CONFIDENCE is an observational study with spontaneous reporting of AEs, a bias in the reporting of AEs, such as underreporting of non-serious AEs and relative overrepresentation of SAEs, cannot be excluded. Furthermore, since immunoglobulin G (IgG) levels were not collected systematically in this study and were only available through spontaneous AE-reporting, no correlation between IgG levels and the prevalence of infections and infestations can be determined.

## 6 Conclusion

Real-world data offer valuable insights into treatment performance outside of controlled clinical trials. The CONFIDENCE population reflects the clinical use of ocrelizumab in Germany (42). Nearly 60% of pwRMS achieved CONFIDENCE treatment success over a treatment duration of 5 years, with higher success rates in early treatment lines.

Over a treatment duration of up to 5.5 years, no new or unexpected AEs were observed, and the number of events did not increase with extended treatment duration. In total, the present findings favor early intervention with ocrelizumab as a high-efficacy treatment over an escalation strategy to optimize long-term outcomes for pwMS.

## Data Availability

The original contributions presented in the study are included in the article/Supplementary material, further inquiries can be directed to the corresponding author.

## Footnotes

^1^ https://catalogues.ema.europa.eu/node/3142/administrative-details
