# Supplementary material for: CONFIDENCE treatment success: long-term real-world effectiveness and safety of ocrelizumab in Germany
Source: Front Neurol. 2025 May 21;16:1564327. doi: 10.3389/fneur.2025.1564327 (PMC12135804; doi:10.3389/fneur.2025.1564327)
Supplement: Supplementary file 1 [file Supplementary_file_1.docx]

Supplementary Material

# Supplementary Tables

**Supplementary Table 1.** Reasons for premature study termination in the CONFIDENCE study (full analysis set).

| n, (%) | **pwRMS (n=2,261)** |
| --- | --- |
| Total number of premature terminations | **409 (**18.1) |
| Reason for premature study termination |  |
| AE | 9 (0.4) |
| Death | 11 (0.5) |
| Lack of efficacy | 6 (0.3) |
| Lost to follow-up | 69 (3.1) |
| Other | 203 (9.0) |
| Physician decision | 15 (0.7) |
| Withdrawal by subject | 96 (4.2) |
| Pregnancy | 3 (0.1) |

One pwRMS can have more than one reason for premature study termination. AE, adverse event; pwRMS, persons with relapsing multiple sclerosis.

**Supplementary Table 2.** Fatal treatment-emergent AEs by MedDRA system organ class and preferred term in the CONFIDENCE study (safety analysis set).

|  | **Total pwRMS (n=2,267)** | **pwRMS by number of PMSTs** | | | |
| --- | --- | --- | --- | --- | --- |
|  |  | **0 PMSTs (n=404)** | **1 PMST (n=548)** | **2 PMST (n=535)** | **≥3 PMSTs (n=780)** |
| **pwRMS with fatal AE, n (%)** | 7 (0.3) | 0 (0.0) | 1 (0.2) | 3 (0.6) | 3 (0.4) |
| **Fatal events, n (%)** | | | | | |
| Cardiac disorders | 3 (0.1) | - | - | 2 (0.4) | 1 (0.1) |
| Acute myocardial infarction | 1 (<0.1) | - | - | 1 (0.2) | - |
| Cardiac disorder | 1 (<0.1) | - | - | - | 1 (0.1) |
| Cardiac failure | 1 (<0.1) | - | - | 1 (0.2) | - |
| Coronary artery disease | 1 (<0.1) | - | - | 1 (0.2) | - |
| Left ventricular failure | 1 (<0.1) | - | - | - | 1 (0.1) |
| Myocarditis | 1 (<0.1) | - | - | - | 1 (0.1) |
| General disorders and administration site conditions | 2 (0.1) | - | - | 2 (0.4) | - |
| Multiple organ dysfunction syndrome | 1 (<0.1) | - | - | 1 (0.2) | - |
| Sudden death | 1 (<0.1) | - | - | 1 (0.2) | - |
| Neoplasms benign, malignant and unspecified (incl cysts and polyps) | 2 (0.1) | - | 1 (0.2) |  | 1 (0.1) |
| Angiosarcoma | 1 (<0.1) | - | 1 (0.2) |  | - |
| Pancreatic carcinoma | 1 (<0.1) | - | - | - | 1 (0.1) |
| Infections and infestations | 1 (<0.1) | - | - | 1 (0.2) | - |
| COVID-19 | 1 (<0.1) | - | - | 1 (0.2) | - |
| Respiratory, thoracic and mediastinal disorders | 1 (<0.1) | - | - | - | 1 (0.1) |
| Pulmonary embolism | 1 (<0.1) | - | - | - | 1 (0.1) |

One pwRMS can have more than one fatal event. AE, adverse event; pwRMS, persons with relapsing multiple sclerosis; PMST, prior multiple sclerosis-specific pre-treatment.

# Supplementary Figures


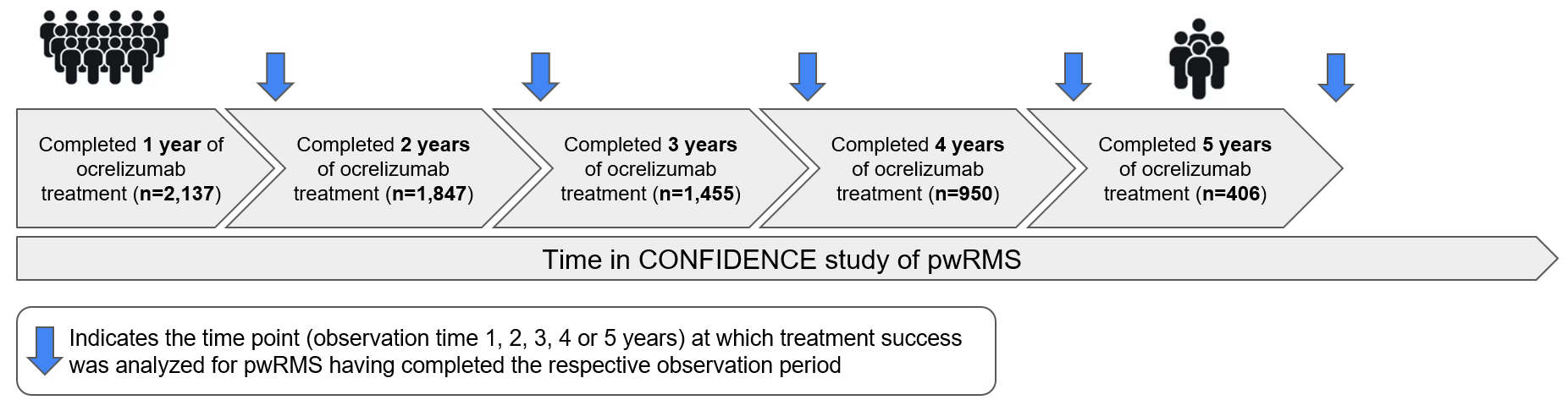


**Supplementary Figure 1.** The concept of landmark analysis for the evaluation of treatment success in CONFIDENCE. In the CONFIDENCE landmark analysis, only pwRMS still on treatment at the end of a specific analysis year were considered for analysis. Therefore, pwRMS who were analyzed for 5 years (n=406) were also included in the analyses for 1, 2, 3 and 4 years, those analyzed for 4 years (n=950) were also included in the analyses for 1, 2 and 3 years etc. Numbers refer to the overall population of the full analysis set. Blue arrows indicate the time points (observation time 1, 2, 3, 4 or 5 years) at which CONFIDENCE treatment success was analyzed for pwRMS having completed the respective observation period. pwRMS, people with relapsing multiple sclerosis.
